# The Influence of Data-Driven Compressed Sensing Reconstruction on Quantitative Pharmacokinetic Analysis in Breast DCE MRI

**DOI:** 10.3390/tomography8030128

**Published:** 2022-06-14

**Authors:** Ping Ni Wang, Julia V. Velikina, Leah C. Henze Bancroft, Alexey A. Samsonov, Frederick Kelcz, Roberta M. Strigel, James H. Holmes

**Affiliations:** 1 Department of Medical Physics, University of Wisconsin-Madison, 1111 Highland Avenue, Madison, WI 53705, USA; b9803216@gmail.com (P.N.W.); rstrigel@uwhealth.org (R.M.S.); 2 Department of Radiology, University of Wisconsin-Madison, 600 Highland Avenue, Madison, WI 53792, USA; velikina@wisc.edu (J.V.V.); lhenze@wisc.edu (L.C.H.B.); samsonov@wisc.edu (A.A.S.); fkelcz@uwhealth.org (F.K.); 3 Carbone Cancer Center, University of Wisconsin-Madison, 600 Highland Avenue, Madison, WI 53792, USA; 4 Department of Radiology, University of Iowa, 169 Newton Road, Iowa City, IA 52333, USA; 5 Holden Comprehensive Cancer Center, University of Iowa, 169 Newton Road, Iowa City, IA 52333, USA

**Keywords:** breast DCE-MRI, compressed sensing, quantitative imaging

## Abstract

Radial acquisition with MOCCO reconstruction has been previously proposed for high spatial and temporal resolution breast DCE imaging. In this work, we characterize MOCCO across a wide range of temporal contrast enhancement in a digital reference object (DRO). Time-resolved radial data was simulated using a DRO with lesions in different PK parameters. The under sampled data were reconstructed at 5 s temporal resolution using the data-driven low-rank temporal model for MOCCO, compressed sensing with temporal total variation (CS-TV) and more conventional low-rank reconstruction (PCB). Our results demonstrated that MOCCO was able to recover curves with K^trans^ values ranging from 0.01 to 0.8 min^−1^ and fixed V_e_ = 0.3, where the fitted results are within a 10% bias error range. MOCCO reconstruction showed less impact on the selection of different temporal models than conventional low-rank reconstruction and the greater error was observed with PCB. CS-TV showed overall underestimation in both K^trans^ and V_e_. For the Monte-Carlo simulations, MOCCO was found to provide the most accurate reconstruction results for curves with intermediate lesion kinetics in the presence of noise. Initial in vivo experiences are reported in one patient volunteer. Overall, MOCCO was able to provide reconstructed time-series data that resulted in a more accurate measurement of PK parameters than PCB and CS-TV.

## 1. Introduction

Dynamic contrast enhanced (DCE) MRI is widely accepted as the most sensitive imaging method for the detection of breast cancer [1,2] and shows promise for assessing response to therapy [3,4,5]. Conventional DCE-MRI protocols using high spatial resolution (at or below 1 mm × 1 mm in-plane pixel size) but low temporal resolution (60–120 s/time-frame) [6] enable evaluation of lesion morphology as well as the kinetic features of lesions based on the MRI BI-RADS lexicon [7]. Kinetic features, including assessment of early wash-in and late wash-out phase images, are used to differentiate between benign and malignant lesions to improve sensitivity and specificity [8]. However, studies have shown that there is an overlap between the kinetic features of benign and malignant lesions with conventional methods [9,10,11,12,13].

To overcome limitations in the specificity of DCE-MRI, prior authors have proposed including quantitative pharmacokinetic (PK) analysis to extract more detailed physiological information from contrast kinetics showing potential for improving diagnostic accuracy and specificity [14,15,16,17]. In breast studies, the extended Tofts model (ETM) is commonly used [18] to provide insight into correlations between tumor angiogenesis and contrast agent (CA) kinetics from the derived PK parameters. For example, the K^trans^ (min^−1^) value is correlated to the wash-in slope describing the transfer rate of CA from plasma to extravascular extracellular space (EES). V_e_ (%) is the volume fraction of EES and V_p_ (%) is the volume fraction of blood plasma. These parameters have shown potential in the evaluation of treatment planning [19], screening [20] and treatment response assessment [21,22], yet their clinical usage has been severely hampered due to the uncertainty of measurement accuracy. 

To accurately measure PK parameters, multiple factors should be considered including the native T_1_ value of the tissue [23], choice of the arterial input function, and temporal resolution of the time-resolved T_1_-weighted imaging sequence [24,25]. Lopata et al. [26] demonstrated that the accuracy of the K^trans^ estimation is highly dependent on the temporal resolution, which becomes increasingly more important for K^trans^ values greater than 0.5 min^−1^. Another simulation study conducted by Giovanni et al. [24] in breast DCE-MRI evaluated the fitting error base on different temporal resolutions showing that the error can be less than 10% for K^trans^ > 0.5 min^−1^ if a temporal resolution of less than 20 s could be achieved. However, advanced acquisition and reconstruction approaches are needed to achieve such high temporal resolution while maintaining the required spatial resolution and large field of view bilateral breast coverage. 

Several accelerated data acquisition approaches have been proposed for dynamic image reconstruction, such as parallel imaging, view-sharing techniques [20,27,28,29,30], low-rank matrix recovery approaches [31] and compressed sensing reconstruction [32,33,34,35]. Parallel imaging alone can only provide moderate acceleration factors and view-sharing techniques can provide higher nominal temporal resolution but have been shown to suffer from temporal blurring [36]. Low-rank matrix recovery approaches assume the dynamic image series can be modeled by a low-dimension subspace, that is only a few temporal basis functions are needed to estimate the kinetic features of each voxel [31]. Studies have exploited the use of rank reduction [37,38] and have shown that a low-rank matrix of the image series can be recovered from under sampled k-space data. However, low-rank techniques are known to suppress temporal dynamics in the cases of complex tissue kinetics, that cannot be accurately represented by a small number of temporal basis functions, especially when high under sampling factors are required. These deficiencies may be overcome by the data-driven model consistency condition (MOCCO) technique proposed by Velikina et al., 2015 that uses low-rank temporal models for regularization instead of hard constraining, which results in full-rank solutions that preserve temporal dynamics [39]. More recently, Wang et al., 2021 optimized using MOCCO for breast DCE-MRI and demonstrated the ability to provide 5 s temporal resolution while still matching the in-plane spatial resolution and full volume coverage typically used in routine clinical protocols [40].

In most studies, the temporal curves generated by using the proposed accelerated techniques were compared with state-of-the-art techniques, such as non-uniform fast Fourier transform (NUFFT) [34,41,42]. However, due to the lack of the ground truth in the in-vivo setting, the temporal accuracy of PK parameters derived from these advanced reconstruction methods has not been studied in a wide range of PK values and scan parameters for bilateral breast DCE-MRI. Simulations using digital reference objects (DROs) provide an important tool for validation purposes due to the ability to provide a ground truth for quantitative analysis. Current community initiatives such as the Radiological Society of North American Quantitative Imaging Alliance (QIBA) propose using DROs to validate the quantitative accuracy of new techniques if these approaches are to be used in clinical practice. 

The aim of this study is to evaluate the accuracy of PK parameter estimation from the data-driven low-rank compressed sensing (MOCCO) reconstruction with 5 s temporal resolution for breast MRI using a range of relevant tissue contrast kinetics and at clinically applicable spatial resolution. Specifically, we will determine how well the MOCCO reconstruction is able to recover relevant slow to rapid contrast kinetics using a breast DCE digital reference object (DRO) with signal characteristics generated using the ETM.

## 2. Materials and Methods

To validate the temporal accuracy of our proposed imaging techniques for estimating quantitative PK parameters, a DRO was used to generate k-space data that included a wide range of tissue contrast kinetics. It was shown that the error of MOCCO reconstruction depends both on the rank of the temporal model and on the ability to learn temporal basis functions from the available data [39]. To evaluate the impact of each source of error on quantitative accuracy, we compared MOCCO reconstructions using temporal models derived from both high and low spatial resolution images. Additionally, Monte-Carlo simulations were performed to evaluate the accuracy and precision of quantitative PK parameters from MOCCO reconstruction in slow and rapid tissue contrast kinetics in the presence of noise. 

### 2.1. Digital Reference Object (DRO)

**Figure 1:**
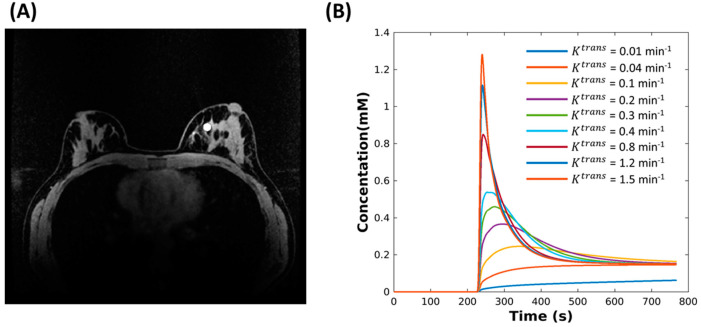
(**A**) A breast digital reference object (DRO) (matrix size 448 × 448 × 142) phantom is shown with one lesion located in the fibroglandular tissue. (**B**) Nine configurations of the DRO phantom were evaluated, each with an 8 mm diameter lesion at the same location and having contrast kinetics generated by assigning the fixed V_e_ = 0.3, fixed V_p_ = 0.001, and K^trans^ ranging from 0.01 to 1.5 using the extended Tofts model, respectively.

The DRO used in this study was recently published by Henze Bancroft et al. [43] and is publicly available through a GitHub repository referenced in their manuscript. The DRO allowed for generation of specific breast tissue structures as well as adding user defined tissue structures with uniquely assigned contrast kinetics (Figure 1). Imaging simulation parameters were chosen for the DRO to replicate a conventional clinical bilateral breast protocol: FOV = 340 mm × 340 mm, in-plane spatial resolution = 0.75 mm × 0.75 mm, slice thickness = 1.4 mm, flip angle = 30°, TE/TR = 2.4 ms/4.7 ms and acquired matrix size = 448 × 448 × 142. Homogeneous round lesions with a diameter of 8 mm were added to simulate enhancing lesions. Note that a flip angle of 30° is not typical for standard clinical breast protocols but is more optimal for PK modeling. The concentration time curves (CTC) for the lesions were modeled using the extended Tofts model (ETM) [44,45]. Parameters for the ETM were chosen to span relatively wide ranges of K^trans^ = 0.01–1.5 min^−1^ and fixed values of $V_{e}=0.3$, and $V_{p}=0.001$. These ranges were selected to extend slightly beyond the typical range of slow, intermediate and rapid changing lesion kinetics to allow performance assessment extending to the limits of the expected parameter ranges. An arterial input function (AIF) curve was simulated by using the publicly available dispersion model described by Barboriak et al. [46]. A hematocrit of 0.45 was assumed. The spoiled gradient recalled echo (SPGR) signal model was then used to generate signal time curves assuming a field strength of 3T, T_1_ value of breast tissue ($T_{10}$ = 1444 ms [47]) and contrast agent relaxivity of $r_{1}$ = 4.9 ${\mathrm{mM}}^{-1}\;s^{-1}$ to simulate Gd-BOPTA (gadobenate dimeglumine, Multihance, Bracco, Milan, Italy) [48], and imaging flip angle (FA = 30°). Images containing both static and dynamic features were then sampled using the NUFFT [49] to simulate k-space data generated from an under sampled golden-angle radial acquisition consisting of 1024 radial projections with a 16-channel breast coil array. The breast coil sensitivity maps were derived from MRI images acquired using a breast shaped water phantom followed by a local fitting method to remove Gibbs ringing artifacts and noise [50]. 

### 2.2. Reconstruction

The under sampled radial data were reconstructed at 5 s temporal resolution corresponding to eight projections per time frame (under sampling factor, R = 88) using MOCCO:

(1) $$\hat{s}=\arg\underset{s}{\min}\left(||Es-{m||}_{2}^{2}+\lambda \left||({\tilde{I}}_{K}{\tilde{I}}_{K}^{*}-I_{t}\right){s||}_{1}\right)$$

here m denotes the measured MRI signal of the underlying image series, which was acquired using an encoding matrix E comprising the coil sensitivity values and Fourier encoding terms. λ is a regularization parameter providing a balance between the data consistency and the regularization terms. ${\tilde{I}}_{K}$ is a matrix of rank K defining the temporal model, whose columns are temporal basis functions learned from the data, and ${\tilde{I}}_{K}^{*}$ is its adjoint matrix. To evaluate the impact of ${\tilde{I}}_{K}$ on reconstruction accuracy, we evaluated two approaches to generate the temporal basis functions for MOCCO:

1. Behavior with temporal models derived from high resolution images (HR): the ${\tilde{I}}_{K}$ for this approach included two elements of the pre-estimated temporal matrix, which included the reference CTCs and temporal curve with constant value to simulate dynamic and static tissue signal changes.
2. Performance with temporal models derived from low resolution images (LR): in this approach, ${\tilde{I}}_{K}$ was learned through the low frequency region from fully-sampled central k-space data using progressive learning with cubic spline approximation [51,52] followed by complex independent component analysis (ICA) [53]. The ICA technique assumes that each component is statistically independent from the source signals, which has been shown to be a robust method to identify key components of the perfusion series and remove unwanted image-to-image fluctuations [54].

MOCCO with ${\tilde{I}}_{K}$ using the HR approach (MOCCO-HR) can provide a theoretical baseline when the ideal temporal model is available. However, MOCCO using the LR approach (MOCCO-LR) presents a more realistic scenario where the temporal model is learned from the data itself. Both MOCCO-HR and MOCCO-LR were implemented using iteratively re-weighted least squares minimization [55]. For comparison, we also applied a more conventional low-rank reconstruction [31,38] approach using a principal component basis (PCB), which assumes that the image series $\hat{s}$ is restricted to a low dimensional subspace, i.e., is of the form

(2) $$\hat{s}\left(x,t\right)={\tilde{I}}_{K}\left(t\right){\tilde{C}}_{K}\left(x\right)$$

where ${\tilde{I}}_{K}\left(t\right)$ is temporal model of rank K and the spatial coefficients ${\tilde{C}}_{K}\left(x\right)$ can be determined by solving a quadratic minimization problem:

(3) $${\tilde{C}}_{K}=\arg\underset{C}{\min}\left(||E{\tilde{I}}_{K}C-{m||}_{2}\right)$$

As with MOCCO, two different temporal models were also used for PCB, denoted as PCB-HR and PCB-LR, and quadratic minimization was implemented using the conjugate gradient method. 

Since individual image pixels often have similar temporal enhancement curves for dynamic imaging, the temporal total variation (TV) is often used as a sparse representation for compressed sensing reconstruction [41,42]. The compressed sensing with temporal total variation (CS-TV) regularization can be defined as

(4) $$\hat{s}=\arg\underset{s}{\min}\left(||Es-{m||}_{2}^{2}+\lambda ||\nabla_{t}{s||}_{1}\right)$$

where $\nabla_{t}$ is the first order temporal gradient.

A regularization value (λ) of 10 and 2 was used for both MOCCO and CS-TV to provide the same visually perceived image sharpness based on previous studies using the same DRO configuration [56]. Iterations of the reconstruction were performed until the relative norm of the k-space residual was less than a specified tolerance (10^−9^) or until a maximal number of iterations (n = 400) was reached for MOCCO, PCB and CS-TV, respectively.

For the purposes of providing reference images for comparison, fully-sampled radial data consisting of 704 individual projections per time frame were generated by matching the temporal resolution of the under sampled radial images. Next, reference images were reconstructed using iterative SENSE reconstruction from fully sampled k-space data.

### 2.3. Analysis

PK fitting was performed to determine how well the original curve shapes were recovered using the radial acquisition and advanced reconstruction methods. Specifically, the fitting was used to measure how well the original kinetic parameters could be recovered from the reconstructed temporal curves. Signal intensity time curves from regions-of-interest (ROI) placed in the lesion locations were measured across all image time-series and subsequently converted to CTCs. PK modeling was performed using the ROCKETSHIP toolbox [56] by fitting the ETM to the CTCs using the Levenberg–Marquardt algorithm with a step tolerance of 1 × 10^−6^ and a function tolerance of 1 × 10^−8^. Fitting bounds were set between 0 and 1 with randomly selected initial estimates for K^trans^, V_e_ and V_p_. The fitted voxel-wise PK parameters were then compared to the original PK parameters to generate % error maps. Bland–Altman plots were used to evaluate the agreement between the PK parameters measured from two different reconstructions and the corresponding ground truth.

Monte-Carlo simulations were performed to evaluate the performance of the MOCCO reconstruction in the presence of noise. Thirty realizations of independent identically distributed (i.i.d.) complex Gaussian noise with zero mean and standard deviation of 20% of the mean k-space magnitude were added to each coil channel for three k-space data sets that included lesions with K^trans^ = 0.01, 0.3 and 1.5 min^−1^ resulting in 90 unique datasets (30 for each tissue contrast kinetics). Additionally, 90 realizations (30 for each tissue contrast kinetics) were performed using fully-sampled data with additive i.i.d. Gaussian noise with the standard deviation matched to the under sampled data. The reconstruction accuracy was assessed by taking a pixel-wise mean and standard deviation across all Monte-Carlo realizations and calculating the percent error between the ground truth and the Monte-Carlo mean from under sampled and fully sampled images, respectively. 

### 2.4. In Vivo Imaging

Two patient volunteers were imaged during contrast injection (gadobenate dimeglumine, Multihance; Bracco Inc., Milan, Italy) on a clinical 3T MRI (Signa Premier, GE Healthcare, Waukesha, WI, USA) using a 16-channel breast coil (Sentinelle, Invivo International, Gainsville, FL, USA) for this institutional review board-approved, HIPPA-compliant study. 

#### MRI Acquisition

Radial imaging was performed using a 3D stack-of-stars golden-angle spoiled gradient echo (SPGR) imaging sequence to sample 1344 unique radial angles. The radial field of view (FOV) was oversampled by doubling the sampling bandwidth to limit aliasing from signal outside the FOV. Acquisition parameters included: repetition time (TR) = 5.87 ms; echo time (TE) = 2.79 ms; FOV = 38 cm; flip angle = 10; receiver bandwidth = +/−63.3 kHz; acquisition matrix = 448 × 448 × 142, acquired spatial resolution = 0.8 × 0.8 mm in-plane resolution and 1.4 mm out of plane, acceleration factor of 1.5 at z phase encoding. A weight-based dose (0.1 mmol/kg) of a gadolinium-based contrast agent (gadobenate dimeglumine, Multihance; Bracco Inc., Milan, Italy) was administered followed by a 20-mL saline flush, both injected at a rate of 2 mL/s using a power injector. 

## 3. Results

**Figure 2:**
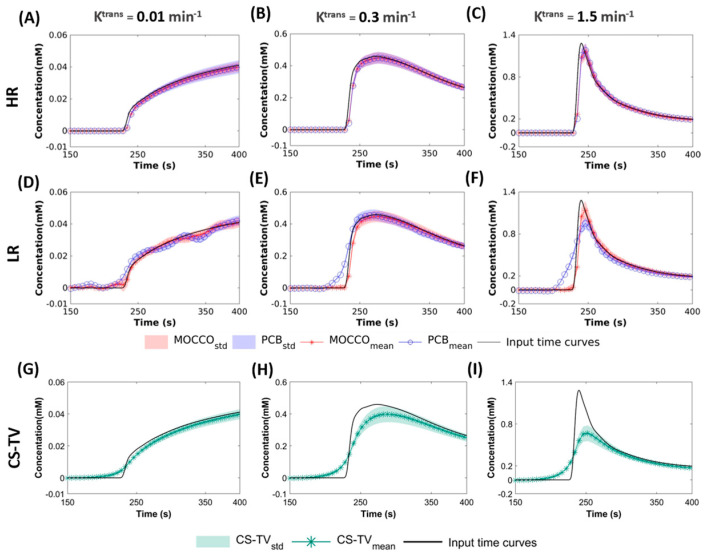
Simulated CTCs with slow (**A**,**D**,**G**), intermediate (**B**,**E**,**H**) and rapid (**C**,**F**,**I**) contrast kinetics in noise-free data (displayed for a subset of time from 150 to 400 s). Mean CTCs measured for three lesions with varying K^trans^ values reconstructed using CS-TV (green star) (**G**–**I**), MOCCO (red star) and PCB (blue circle) with the temporal model derived from high spatial resolution (HR) (**A**–**C**) and low spatial resolution (LR) images (**D**–**F**). The corresponding standard deviations within the lesions are shown with banded area. The input time curves (“truth”) used to generate the source data are plotted with dark black lines in all frames.

Figure 2 illustrates the temporal performance of MOCCO-HR, MOCCO-LR, PCB-HR PCB-LR and CS-TV for recovering the simulated lesion CTCs with varying enhancement patterns. Note that the displayed time interval ranges from 150 s to 400 s to allow for a better visualization over the period of greatest signal change. The HR approach with K = 2 represents the best approximation of lesion kinetics for the reconstructions when using the reference CTCs as the temporal basis. Therefore, as illustrated in Figure 2A–C, the mean concentration value of the temporal curves generated by both PCB-HR and MOCCO-HR are closely matched to the original reference time curves in the noise-free dataset. For the LR approach with K = 3 in Figure 2D–F, MOCCO-LR showed similar results to the MOCCO-HR with only slightly increased standard deviation whereas PCB-LR has shown severe temporal blurring on wash-in slopes in both intermediate (Figure 2E) and rapid (Figure 2F) contrast kinetics. CS-TV shows general over-smoothing of the temporal curves across the different lesion kinetics (Figure 2G–I). 

**Figure 3:**
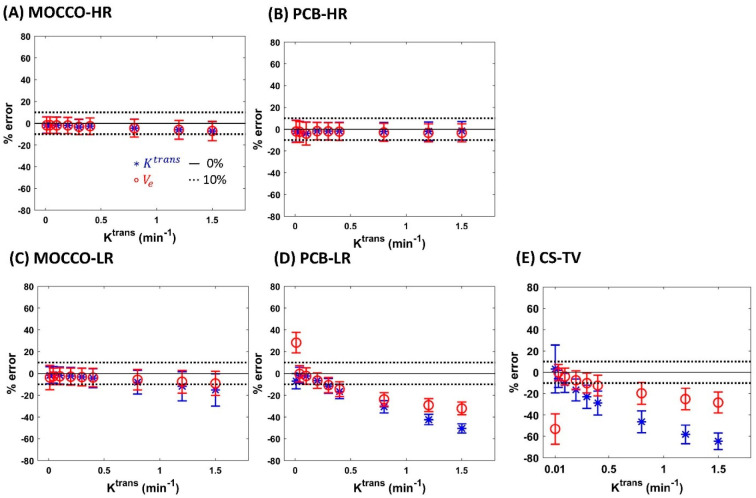
The influence of temporal model in noise-free data using MOCCO (**A**,**C**), PCB (**B**,**D**) and CS-TV (**E**) reconstruction on parameter estimation of K^trans^ and V_e_. Bland–Altman plots show the mean (±standard deviation) of K^trans^ (blue stars) and V_e_ (red circles). The ±10% error range is shown as black dashed lines. Note the results for the HR approaches (**A**,**B**) represent idealized scenarios where the full spatial resolution temporal model can be utilized whereas the LR approaches (**C**,**D**) represent more realistic scenarios where the temporal model could be learned from the under sampled data.

**Figure 4:**
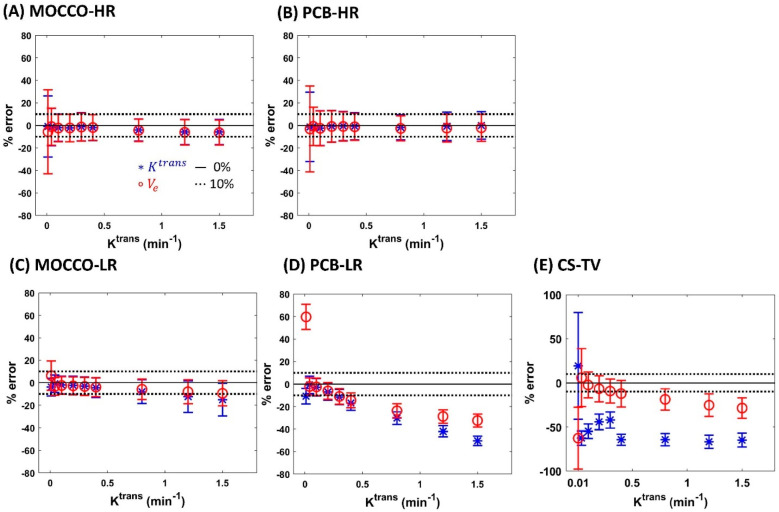
The influence of temporal model and noise using MOCCO (**A**,**C**), PCB (**B**,**D**) and CS-TV (**E**) reconstruction on parameter estimation of K^trans^ and V_e_. Bland–Altman plots show the mean (±standard deviation) of K^trans^ (blue stars) and V_e_ (red circles). The ±10% error range is shown as black dash lines.

Bland–Altman plots of the fitted K^trans^ and V_e_ within the lesion ROI in noise-free data and in data with 20% noise added are shown in Figure 3 and Figure 4, respectively. In noise-free data, the fitted K^trans^ and V_e_ were within a 10% bias error range of the corresponding ground truth K^trans^ and V_e_ for MOCCO-HR (Figure 3A) and PCB-HR (Figure 3B). The results from MOCCO-LR (Figure 3C) were aligned with MOCCO-HR (Figure 3A) for CTC with K^trans^ < 0.8 min^−1^. For the cases with K^trans^ ≥ 0.8 min^−1^, the error range was close to or slightly larger than the limits of the 10% error range. On the contrary, the PCB-LR (Figure 3D) and CS-TV (Figure 3E) showed much greater underestimation of the fitted K^trans^ and V_e_ (errors exceeding the 10% range) when K^trans^ ≥ 0.3 min^−1^ and 0.2 min^−1^, respectively.

In data with 20% noise added, the CTCs with K^trans^ = 0.01 and 0.04 in MOCCO-HR (Figure 4A) and PCB-HR (Figure 4B) showed greater increased standard deviation within the lesions, whereas similar results of the mean % error reconstructed by MOCCO-LR (Figure 4C) and PCB-LR (Figure 4D) were observed compared with the results in noise-free data (Figure 3C,D). On the contrary, CS-TV (Figure 4E) showed overall increased error across all fitted K^trans^ values compared with the results in noise-free data (Figure 3E). A larger increased mean error was observed at the K^trans^ ≤ 0.2 min^−1^. Note that the same reconstruction parameters were used to reconstruct both the noise-free and the corresponding noisy-data. 

**Figure 5:**
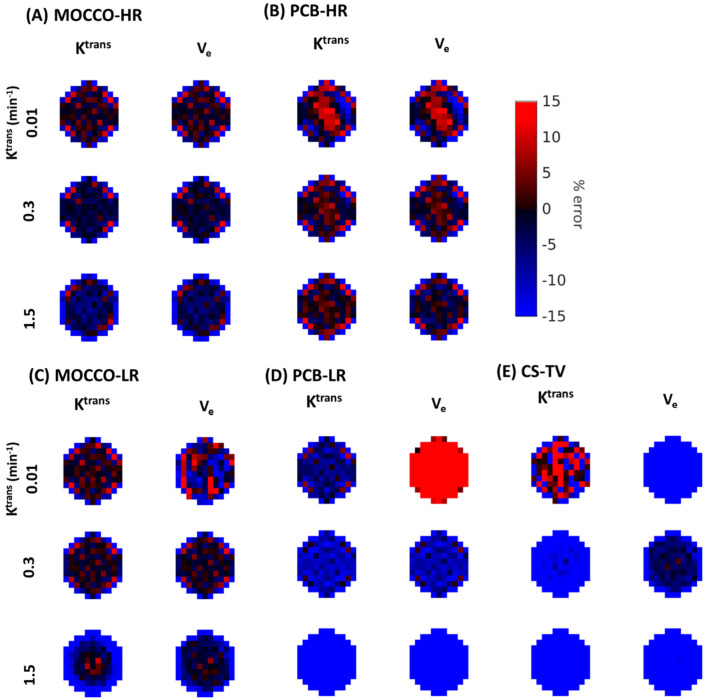
Visualization of zoomed-in error maps for K^trans^ and V_e_ from (**A**) MOCCO-HR, (**B**) PCB-HR, (**C**) MOCCO-LR, (**D**) PCB-LR and (**E**) CS-TV without noise added to the simulated lesions with K^trans^ = 0.01, 0.3, 1.5 min^−1^, obtained by measuring the % differences between the fitted parameters and the true values for the lesion.

**Figure 6:**
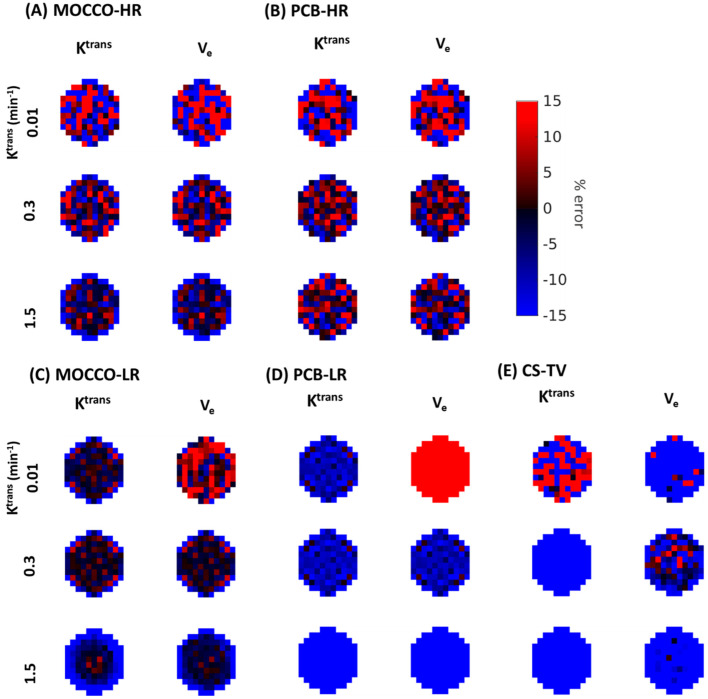
Visualization of zoomed-in error maps for K^trans^ and V_e_ from (**A**) MOCCO-HR, (**B**) PCB-HR, (**C**) MOCCO-LR, (**D**) PCB-LR and (**E**) CS-TV with 20% noise added to the simulated lesions with K^trans^ = 0.01, 0.3, 1.5 min^−1^, obtained by measuring the % differences between the fitted parameters and the true values for the lesion.

In order to better assess the source of the signal differences within the lesion including the increased standard deviation observed with some of the reconstructions, spatial mapping of the PK fitting results was performed. Figure 5 and Figure 6 demonstrate the percent difference zoomed-in error maps of the derived K^trans^ and V_e_ from MOCCO and PCB without and with 20% noise added, respectively. Color maps of the lesion with K^trans^ = 0.01, 0.3 and 1.5 were selected to show the difference between the slow, intermediate and rapid contrast kinetics with and without noise added. In results of noise-free data, the zoomed-in error map from MOCCO-HR (Figure 5A) showed relatively homogenous under-estimation of K^trans^ and V_e_ in the error distribution for the intermediate and rapid contrast kinetics. Only results from the lesion with slow contrast kinetics (K^trans^ = 0.01 min^−1^) displayed a mixed response with over- and under-estimation of both K^trans^ and V_e_ with an error range within ±10%. MOCCO-LR (Figure 5C) demonstrated similar error distribution compared with MOCCO-HR, with only slightly increased error observed at the lesion edge for rapid contrast kinetics (K^trans^ = 1.5 min^−1^) in error map of K^trans^ and V_e_. In contrast, there is a discrepancy in the error distributions between PCB-HR (Figure 5B) and PCB-LR (Figure 5D) without the noise added. The error maps derived from PCB-HR (Figure 5B) displayed more heterogeneous over- and under-estimation over the entire lesion but resulted in lower % error across all three lesions for both K^trans^ and V_e_, which results in lower mean error but higher standard deviation (note that the % mean and standard deviation are shown in Table 1). Table 1 also includes a comparison against the simulated setting of acquiring a fully sampled dataset at 5 s temporal resolution to demonstrate the level of error introduced through the PK modeling at this discrete time-sampling. The PCB-LR (Figure 5D) demonstrated a relatively homogeneous error distribution within the lesion but showed an overall increased under-estimation of both K^trans^ and V_e_. Only the error maps of V_e_ with K^trans^ = 0.01 min^−1^ showed overestimation of the entire lesion. 

In data with 20% noise added, an increased deviation in the percent error of both K^trans^ and V_e_ was observed in MOCCO-HR (Figure 6A) and PCB = HR (Figure 6B) but no major difference was observed in MOCCO-LR (Figure 6C) and PCB-LR (Figure 6D). Increased error was only observed in the error maps of V_e_ with K^trans^ = 0.01 min^−1^.

**Figure 7:**
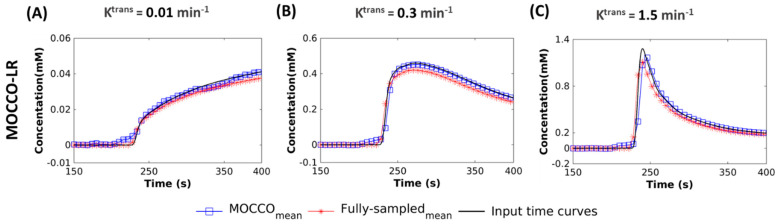
Simulated CTCs with 20% noise added (displayed for a subset of time from 150 s to 400 s). Mean CTCs measured for three lesions with K^trans^ values of 0.1 min^−1^ (**A**), 0.3 min^−1^ (**B**) and 1.5 min^−1^ (**C**). values reconstructed using MOCCO-LR. The input time curves (“truth”) used to generate the source data are plotted with dark black lines in all frames.

Figure 7 shows the temporal curves with K^trans^ = 0.01, 0.3 and 1.5 min^−1^ obtained from MOCCO-LR and the corresponding fully-sampled images by measuring the mean across the Monte-Carlo realizations with noise levels of 20%. The results demonstrated that the mean signal values were found to closely match the curves from the ground truth with very small standard deviation.

**Figure 8:**
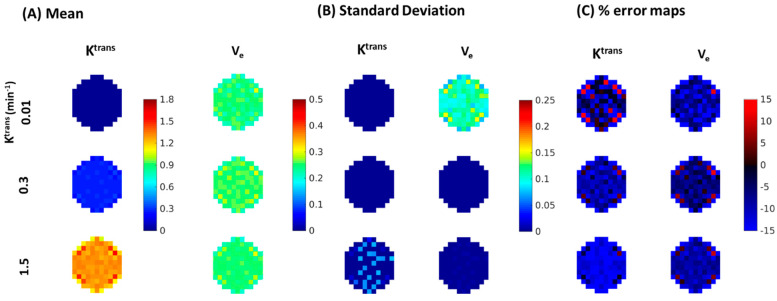
Visualization of zoomed-in color maps for K^trans^ and V_e_ from fully-sampled data with 20% noise added to K^trans^ = 0.01, 0.3, 1.5 min^−1^ obtained by measuring the (**A**) mean, (**B**) standard deviation, (**C**) percent differences between the fitted parameters from all Monte-Carlo noise realizations and the true values for the lesions.

**Figure 9:**
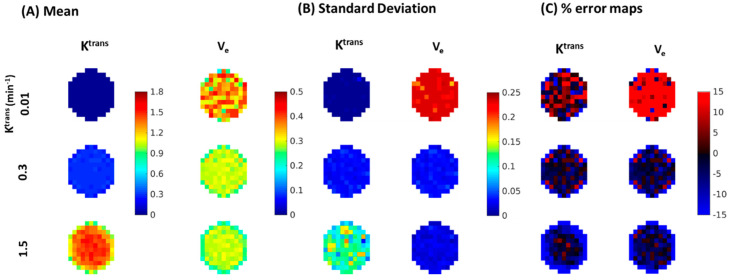
Visualization of zoomed-in color maps for K^trans^ and V_e_ from MOCCO-LR with 20% noise added to K^trans^ = 0.01, 0.3, 1.5 min^−1^ obtained by measuring the (**A**) mean, (**B**) standard deviation, (**C**) percent differences between the fitted parameters from all Monte-Carlo noise realizations and the true values for the lesions.

Figure 8 depicts the results of the Monte-Carlo simulation in estimating the mean (Figure 8A), standard deviation (Figure 8B) and percent error between the Monte-Carlo mean and the true values for lesions (Figure 8C) from MOCCO-LR images. Corresponding results are shown for the fully sampled data in Figure 9. MOCCO-LR was found to provide the most accurate reconstruction results for curves with the middle value of K^trans^ = 0.3 min^−1^, where the overall performance was consistent with the fully-sampled dataset. Only slightly higher standard deviation (~5%) was found in MOCCO-LR for the Monte-Carlo simulation including noise. For curves with slow contrast kinetics (K^trans^ = 0.01 min^−1^), there was no obvious difference between MOCCO-LR and the fully sampled data in the mean and standard deviation of K^trans^ on visual inspection. However, increased variations in estimates of V_e_ were observed. For curves with fast contrast kinetics (K^trans^ = 1.5 min^−1^), the V_e_ value was recovered with low reconstruction error. Although MOCCO-LR showed relatively accurate measurements of the mean K^trans^ values, increased standard deviation was observed in the Monte-Carlo simulation. 

**Figure 10:**
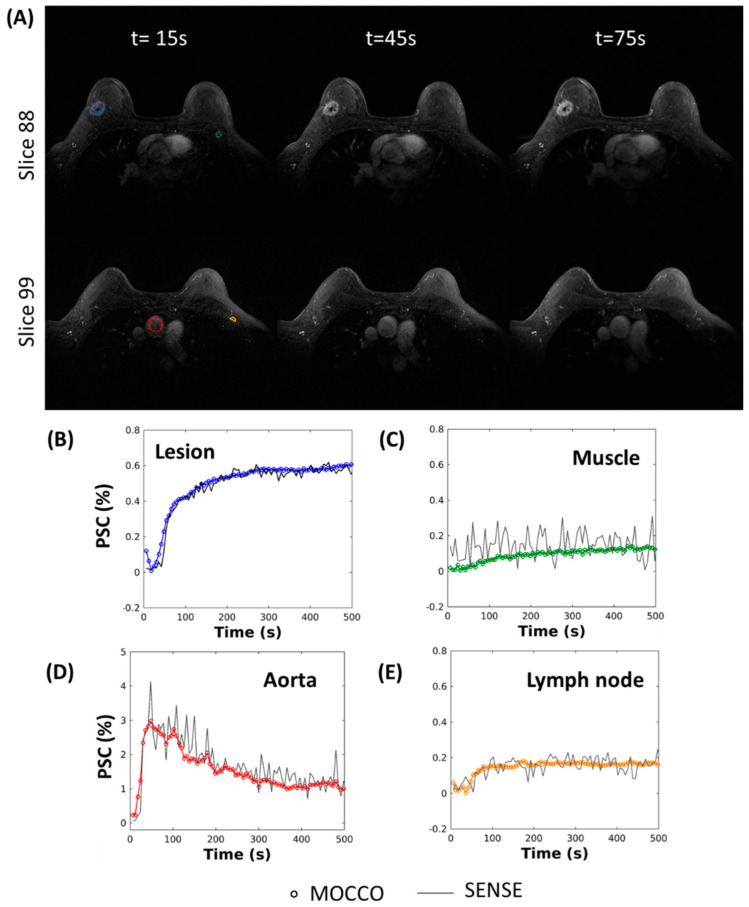
Time-resolved DCE images from a patient volunteer reconstructed using MOCCO-LR with 5 s temporal resolution (**A**). Curves of the percent signal change (PSC) are plotted from ROIs placed in the lesion ((**B**), blue), muscle ((**C**), green), aorta ((**D**), red) and lymph node ((**E**), yellow).

Figure 10 demonstrates the in vivo results for a patient volunteer with an enhancing lesion, using the radial acquisition with MOCCO-LR. High image quality is observed along all time frames. Rapid wash-in and wash-out contrast kinetics are observed in the aorta (Figure 10D). The enhancing lesion showed relatively rapid contrast uptake (Figure 10B), while slower contrast update was observed in the pectoralis muscle and a contralateral lymph node (Figure 10C,E).

## 4. Discussion

In this work, we present a framework to evaluate the temporal fidelity of our proposed technique, the combination of golden-angle stack-of-stars radial acquisition with a data-driven low-rank based CS reconstruction (MOCCO), using quantitative PK analysis for breast DCE-MRI. The DRO used in this study can be employed to simulate a wide spectrum of tissue contrast kinetics with a user-defined PK model and different levels of noise in the data. This simulation approach provides the opportunity to validate the accuracy of our proposed technique for quantitative analysis, which can be difficult to achieve in patient studies. MOCCO reconstruction was also compared to another low-rank method (PCB) as well as a more general CS-based reconstruction algorithm that uses temporal total variation as a sparsity transform (CS-TV) to assess the performance with respect to the selection of temporal model and in the setting of noisy data. 

We present results from generating the temporal basis functions using ICA on low spatial resolution images (LR) and benchmark these against using ICA on high resolution images (HR). The results from HR provide a lower bound on the reconstruction accuracy of MOCCO in the theoretical setting where the exact temporal model can be learned. This idealized scenario resulted in only a small error when compared with reference CTCs that were simulated to reflect the non-realistic case of achieving full angular sampling over all phase encodes at a 5 s temporal resolution. The reference CTCs demonstrate one of the strengths of the digital reference objects in that it is possible to simulate temporal sampling rates well beyond those that can be achieved on current state of the art hardware. The LR approach learned the temporal model from the under sampled data, which reflects real-life scenarios and has been demonstrated to be an effective approach in many other applications [38,39]. In our simulation results, MOCCO-LR produced time-resolved images with high temporal fidelity that enabled robust and consistent estimation of K^trans^ and V_e_ compared with MOCCO-HR. On the other hand, we observed increased errors using PCB-LR, especially in lesions with the highest K^trans^ value (1.5 min^−1^). This can be explained by the fact that MOCCO was shown to produce high-rank solutions even using low-rank temporal models [39], whereas the PCB approach limits the reconstruction result to a low-dimensional subspace, which may be inadequate for describing complex contrast dynamics. In our simulations, MOCCO was found to be less sensitive to the selection of the temporal model and led to a more stable solution, which is consistent with the conclusion from prior works [39,56].

We have demonstrated the comparison between two different CS-based reconstruction approaches that use different temporal models. The selection of the reconstruction regularization parameters for both reconstructions was chosen to optimize the image sharpness and resolution to avoid loss of fine imaging features and small lesions. The results showed that the error range of the fitted PK parameters using the signal-specific temporal model (MOCCO) was improved as compared to the use of a generic sparsity transform in the form of the temporal TV approach (CS-TV). These results were aligned with prior work showing that the MOCCO technique could outperform CS-TV with improved temporal fidelity when matching the spatial resolution and coverage from routine clinical protocols [40]. 

The PK parameters obtained from tissue CTCs with very low K^trans^ < 0.04 min^−1^ were found to be vulnerable to noise for both MOCCO-HR and MOCCO-LR. This is attributed to the low intensity of signal enhancement that can be easily corrupted by the background noise. Otherwise, both techniques were found to be less affected by noise in the source data with K^trans^ > 0.04 min^−1^ and provided similar measurements of PK parameters to the results in the noise-free simulations. The Monte-Carlo simulations also demonstrated that MOCCO-LR was less impacted by noise in the source data and provided better reconstruction accuracy with K^trans^ above 0.3 min^−1^ that is typically of greater interest for characterizing suspicious lesions. 

In our simulations, we found that the increased reconstruction error in the low contrast kinetics (K^trans^ = 0.01 min^−1^) was mostly due to the overall lower maximum lesion CTC peak amplitude. This effect was demonstrated by increased standard deviation in estimations of V_e_ including for lesions measured from the fully sampled data, however slower contrast kinetics are usually of less clinical interest. 

Although multiple studies have proposed new imaging methods to improve the spatial and temporal resolution for breast DCE-MRI, few studies have investigated absolute quantification of the derived PK parameters. One of the challenges is the lack of known ground truth measurements of the contrast signal kinetics due to the need for high temporal and 3D spatial resolution as well as confounding effects such as physiological variability, motion, $B_{0}$ and $B_{1}$ inhomogeneities and accurate tissue T_1_ values. The DRO used in this study allowed for simulating a wide spectrum of known ground truth contrast kinetics to compare reconstruction techniques and characterize performance. In this study, we focus on the impact of temporal accuracy when achieving under 5 s temporal resolution, which shows the potential to provide robust quantitative PK parameters and can be applied to different clinical settings, such as differentiating lesion types and evaluating response to neoadjuvant chemotherapy [21,22].

There are some limitations to the current study. We have shown that accuracy of the PK parameters estimated with both PCB and, to a lesser degree, MOCCO depend on the availability of an adequate temporal model. However, it may be more challenging to obtain such temporal models in situations with highly under sampled data and/or in the presence of motion, based on our current strategy. Therefore, further work is needed to investigate obtaining temporal models using other techniques (e.g., dictionary learning [57]). In this study, emphasis was placed on the evaluation of temporal fidelity of MOCCO reconstruction, and therefore populated AIF curves were used. Although population-based AIFs are still routinely used due to the inability to achieve sufficiently high temporal and spatial resolution to resolve the extremely rapid kinetics, these do represent a limitation to the ability to accurately model a given individual perfusion setting. We have demonstrated the feasibility of using radial acquisition with MOCCO reconstruction to achieve temporal resolution of 5 s at a clinically relevant spatial resolution of 0.8 × 0.8 mm. Future work will aim to validate these results through larger patient studies in the setting of breast cancer.

## 5. Conclusions

We have evaluated the temporal fidelity of the data-driven low-rank compressed sensing reconstruction (MOCCO) reconstruction for recovering a wide range of PK parameters and in the presence of noise to better match typical in vivo settings. Results from the more practical scenarios of learning the temporal model (LR) using low spatial frequency data were compared to the theoretical idealized scenario where the exact temporal model can be learned (HR). We have demonstrated that using MOCCO reconstruction for an image series at a temporal resolution of 5 sec and spatial resolution of 0.8 mm × 0.8 mm × 1.2 mm would lead to an error with 10% or less for V_e_ across all K^trans^ values and an error of −0.9% to −10% for K^trans^ values of less than 0.8 min^−1^ in this DRO simulation matching the clinical setting for DCE-MRI. Only contrast kinetics with very high K^trans^ values beyond the typical in vivo range showed larger errors. Overall, MOCCO was able to provide a reconstructed time-series that resulted in a more accurate measurement of PK parameters than the general low-rank technique (PCB) as well as a more general CS-based reconstruction algorithm that uses temporal total variation as a sparsity transform (CS-TV).

## Figures and Tables

**Table 1:** Summary of results and % errors for K^trans^ and V_e_ from PK model fitting to three different reconstruction results without and with 20% noise added. Note that red text indicates errors with magnitude greater than 10%.

		Reference	MOCCO-HR		PCB-HR		MOCCO-LR		PCB-LR		CS-TV	
% error for K^trans^	**K^trans^**	**0%**	**0%**	**20%**	**0%**	**20%**	**0%**	**20%**	**0%**	**20%**	**0%**	**20%**
	0.01	−4.7	−1.65 ± 7.52	−0.99 ± 27.14	−1.79 ± −7.04	−1.32 ± 30.77	−1.74 ± 7.81	−3.91 ± 7.87	−7.04 ± 7.02	−10.77 ± 6.84	3.17 ± 22.67	19.24 ± 60.41
	0.04	0.06	−1.72 ± 7.49	−1.46 ± 16.54	−2.36 ± −1.69	−0.80 ± 17.04	−1.21 ± 7.67	−0.92 ± 7.75	−1.69 ± 7.5	−0.68 ± 7.71	−6.15 ± 8.06	−62.50 ± 8.11
	0.1	0.09	−1.72 ± 7.51	−2.03 ± 12.07	−3.96 ± −2.3	−2.40 ± 15.48	−1.83 ± 7.78	−2.07 ± 7.85	−2.3 ± 7.52	−2.79 ± 7.74	−9.94 ± 9.05	−54.77 ± 8.11
	0.2	−0.13	−2.04 ± 7.47	−2.22 ± 12.2	−1.64 ± −6.54	−0.70 ± 13.96	−2.38 ± 8.02	−2.33 ± 8.1	−6.54 ± 7.13	−6.94 ± 7.3	−16.52 ± 10.29	−44.29 ± 9.00
	0.3	−1.50	−3.53 ± 6.9	−1.15 ± 12.49	−1.72 ± −11.51	−0.59 ± 12.98	−3.22 ± 8.19	−3.09 ± 8.17	−11.51 ± 6.86	−11.5 ± 6.84	−22.92 ± 10.95	−42.09 ± 9.20
	0.4	−1.91	−2.7 ± 7.55	−1.97 ± 11.43	−1.87 ± −16.58	−0.72 ± 12.05	−4.35 ± 8.64	−4.45 ± 8.74	−16.58 ± 6.59	−16.79 ± 6.46	−28.79 ± 11.11	−64.42 ± 6.31
	0.8	−2.71	−4.39 ± 8.16	−4.04 ± 9.86	−2.13 ± −30.54	−1.67 ± 11.21	−8.07 ± 10.85	−7.94 ± 10.65	−30.54 ± 5.6	−30.43 ± 5.67	−46.46 ± 10.21	−64.26 ± 6.90
	1.2	−3.64	−6.03 ± 8.55	−5.83 ± 11.23	−1.90 ± −42.34	−0.80 ± 12.58	−11.76 ± 13.32	−12.49 ± 13.97	−42.34 ± 4.86	−42.04 ± 4.88	−58.19 ± 8.68	−66.76 ± 7.46
	1.5	−4.86	−7.17 ± 8.76	−5.81 ± 11.19	−1.48 ± −50.6	−0.05 ± 12.23	−15.1 ± 14.86	−15.04 ± 14.54	−50.6 ± 4.28	−50.44 ± 4.27	−64.58 ± 7.63	−64.75 ± 7.90
% error for V_e_	**K^trans^**	**0%**	**0%**	**20%**	**0%**	**20%**	**0%**	**20%**	**0%**	**20%**	**0%**	**20%**
	0.01	−8.38	−1.86 ± 7.55	−5.65 ± 37.29	−2.03 ± 10.10	−3.18 ± 38.12	−3.71 ± 11.14	6.22 ± 13	28.27 ± 9.47	59.68 ± 11.16	−53.17 ± 14.28	−64.65 ± 35.00
	0.04	−0.36	−1.74 ± 7.49	−1.07 ± 16.49	−2.44 ± 9.49	−0.80 ± 17.04	−2.72 ± 7.68	−3.37 ± 7.77	−0.35 ± 7.59	−2.09 ± 7.59	−0.12 ± 7.53	5.86 ± 32.90
	0.1	−0.08	−1.9 ± 7.51	−2.35 ± 12.11	−4.12 ± 10.46	−2.60 ± 15.44	−2.65 ± 7.68	−2.51 ± 7.82	−2.36 ± 7.51	−2.46 ± 7.76	−3.92 ± 7.82	−2.45 ± 14.76
	0.2	−0.13	−1.95 ± 7.5	−2.28 ± 12.25	−1.79 ± 8.02	−0.96 ± 13.92	−2.98 ± 7.86	−2.81 ± 7.88	−6.56 ± 7.12	−6.05 ± 7.37	−7.40 ± 8.69	−6.70 ± 14.90
	0.3	−0.73	−3.24 ± 7.02	−1.44 ± 12.38	−1.93 ± 7.95	−0.90 ± 12.94	−3.34 ± 8.03	−3.36 ± 8.04	−10.67 ± 6.93	−10.96 ± 6.88	−10.07 ± 9.31	−9.32 ± 13.66
	0.4	−0.95	−2.66 ± 7.59	−1.88 ± 11.39	−2.23 ± 8.14	−1.22 ± 12.00	−3.79 ± 8.39	−3.91 ± 8.49	−14.4 ± 6.77	−14.43 ± 6.64	−12.43 ± 9.69	−12.31 ± 15.04
	0.8	−1.42	−4.48 ± 8.15	−4.34 ± 9.93	−3.00 ± 8.28	−2.68 ± 11.09	−5.78 ± 9.37	−5.86 ± 9.14	−23.77 ± 6.15	−23.73 ± 6.22	−19.72 ± 10.23	−18.81 ± 12.10
	1.2	−1.67	−6.04 ± 8.59	−6.13 ± 11.23	−3.34 ± 8.31	−2.41 ± 12.36	−7.57 ± 10.45	−8.02 ± 10.58	−29.09 ± 5.98	−28.93 ± 5.99	−25.12 ± 10.53	−25.39 ± 12.70
	1.5	−1.87	−7.06 ± 8.86	−6.39 ± 11.06	−3.37 ± 8.25	−2.10 ± 12.00	−9.13 ± 11.03	−9.45 ± 11.13	−32.13 ± 5.89	−32.57 ± 5.82	−28.38 ± 9.78	−28.62 ± 11.57

## Data Availability

The code for the digital phantom used in this study is publicly available at https://github.com/lchenze/DRO_Breast_DCE_MRI, accessed on 31 May 2022.
